# Investigation of drug resistance of caries-related streptococci to antimicrobial peptide GH12

**DOI:** 10.3389/fcimb.2022.991938

**Published:** 2022-09-08

**Authors:** Xinwei Li, Yufei Wang, Xuelian Jiang, Yuhao Zeng, Xinran Zhao, Jumpei Washio, Nobuhiro Takahashi, Linglin Zhang

**Affiliations:** ^1^ State Key Laboratory of Oral Diseases & National Clinical Research Center for Oral Disease, West China Hospital of Stomatology, Sichuan University, Chengdu, China; ^2^ Department of Stomatology, Chengdu Second People’s Hospital, Chengdu, China; ^3^ Division of Oral Ecology and Biochemistry, Graduate School of Dentistry, Tohoku University, Sendai, Japan

**Keywords:** antimicrobial peptide, drug resistance, dental caries, streptococci, whole genome sequencing

## Abstract

Dental caries is associated with caries-related streptococci and antimicrobial agents have been widely used for caries control, but troubled by antibiotic resistance. This study aimed to investigate the intrinsic and acquired resistance of caries-related streptococci to antimicrobial peptide GH12, which was proven promising for caries control, and preliminarily explore the phenotypic changes and whole genome of stable acquired resistant strains. In this study, susceptibility assays and resistance assays were performed, followed by stability assays of resistance, to evaluate the intrinsic resistance and the potential resistance of caries-related streptococci. Then, the phenotypic changes of the stable acquired resistant strain were explored. The whole genome of the resistant strain was sequenced and analyzed by second-generation and third-generation high-throughput sequencing technologies. *Streptococcus gordonii* and *Streptococcus sanguinis* were intrinsically resistant to GH12 compared to cariogenic *Streptococcus mutans*. Acquired GH12 resistance in one *S. sanguinis* and four *S. mutans* clinical strains was transient but stable in one *S. mutans* strain (COCC33-14). However, acquired resistance to daptomycin (DAP) and chlorhexidine in all strains was stable. Furthermore, the COCC33-14 showed cross-resistance to DAP and delayed growth rates and a lower population. However, no drug-resistant gene mutation was detected in this strain, but 6 new and 5 missing genes were found. Among them, annotation of one new gene (gene 1782|COCC33-14R) is related to the integral component of the membrane, and one missing gene *rpsN* is associated with the metabolism and growth of bacteria. The results indicate that stable resistant mutants of caries-related streptococci could hardly be selected by exposure to consecutive sublethal GH12, but the risk still existed. Resistance in COCC33-14R is mainly related to changes in the cell envelope.

## Introduction

Dental caries affects approximately 3.9 billion people, causing huge health and economic burdens (Pitts et al., 2017), and is directly associated with biofilm communities, in which several oral streptococci, such as *Streptococcus mutans*, *Streptococcus gordonii* and *Streptococcus sanguinis*, are involved in the caries process (Takahashi and Nyvad, 2011).Among them, *S. gordonii* and *S. sanguinis* are considered more related to symbiotic or health-associated oral conditions, while *S. mutans* is considered more cariogenic especially in the progress of caries (Takahashi and Nyvad, 2011; Wright, 2011).

As dental caries is closely associated with oral bacteria, antimicrobial agents are widely used and studied in the prevention and treatment of caries. Fluoride has been introduced to caries control since 1980s (Buzalafa et al., 2011), and made huge contribution to the decline of caries in recent decades. It has been used in community water fluoridation, toothpaste in general population (Walsh et al., 2019; Whelton et al., 2019) and varnish in high caries risk children (Carey, 2014). According to World Oral Health report 2003 (Petersen and Lennon, 2004), fluoride made extraordinary effectiveness in caries prevention of all ages. One community-randomized controlled trial in dental caries among Australian Aboriginal children demonstrated that F-varnish application once every 6 months for 2 years significantly reduce adjusted d_3_mfs increment in the intervention group (Slade et al., 2011). CHX was one of the first antiseptic agents proposed for dental caries and has proved to be effective for retardation of smooth caries with dentifrice containing 1% chlorhexidine (Gjermo, 1974; Johansen et al., 1975). CHX varnish could reduce the mean number of new white spot lesions of orthodontic patients (Lipták et al., 2018), and improve oral hygiene of high caries risk patients such as geriatric and xerostomia patients and post head and neck radiation patients (Slot et al., 2011; Hong et al., 2018). Hong *et al.* (Hong et al., 2018) showed that once daily regimens of 0.12% chlorhexidine in post head and neck radiation patients contributed to significant reduction in plaque index and decrease in salivary *S. mutans* count. Quaternary ammonium salts (QAS) was added into mouthwash in 1970s and was proved effective to reduce dental plaques (Ciancio et al., 1975). Nowadays, different forms of QAS were successfully added into dental materials, such as dental adhensive (Melo et al., 2013) and resin composites (Cheng et al., 2016) to prevent secondary caries. An adhesive containing dimethylaminododecyl methacrylate (DMADDM) significantly reduced both lesion depth and mineral loss, and also lowered the Keyes scores *in vivo* in a secondary caries animal model (Wu et al., 2018).

However, there are some arguments that resistant strains could emerge during frequent use and drug misuse. Antimicrobial resistance (AMR) in pathogenic bacteria has been a worldwide problem, causing enormous economic loss and a global health crisis (Wright, 2011). Bacteria can be intrinsically resistant to certain antibiotics and can obtain acquired resistance through long-term selective pressure. Intrinsic resistance of bacteria to a particular antibiotic is the ability to resist the action of that antibiotic due to inherent structural or functional properties (Blair et al., 2015). Acquired resistance is generally mediated by genetic mutation in chromosomes or *via* horizontal gene transfer (Munita and Arias, 2016). Acquired resistance with the acquisition of genetic determinants has the highest risk of horizontal spread and the most unacceptable harm.

Oral streptococci have shown an increased frequency of AMR, such as clindamycin (Loyola-Rodriguez et al., 2018), tetracycline (Diaz-Torres et al., 2006), amoxicillin (Diaz-Torres et al., 2006), penicillin (Pasquantonio et al., 2012), fluoroquinolones (De Lastours and Fantin, 2015) and *lipopeptide* daptomycin (DAP) (Garcia-de-la-Maria et al., 2013). In addition, AMR have been found under topical antimicrobial agent applications, such as fluoride (Liao et al., 2017) and CHX (Suppiger et al., 2020). Since oral streptococci are the dominant part of dental plaques and initially attach to the tooth surface (Lee and Lee, 2019), the resistance of caries-related streptococci needs to be studied preferentially.

Antimicrobial peptides (AMPs) are attracting interest worldwide and are generally considered as alternative therapy of conventional antibiotics. The bactericidal mechanisms of AMP are considered to involve cationic AMP that is adsorbed on the anionic bacterial outer membrane and pore formation resulting in general membrane disruption (Lee et al., 2016). However, AMP resistance has arisen through their clinical use, such as polymycin (Moffatt et al., 2019), colistin (Fernandez and Hancock, 2012) and DAP. DAP is an antimicrobial *lipopeptide* that has been applied in the clinic since 2003 (Blair et al., 2015) for multiresistant gram-positive bacteria (Larioza et al., 2011). Unfortunately, some bacteria developed resistance toward DAP during these years, including Mitis group streptococci (Garcia-de-la-Maria et al., 2013; Parrett et al., 2020) and *Staphylococcus aureus* (Kaatz et al., 2006). In the field of caries prevention and control, synthetic or natural AMPs, such as defensins and derivatives (Aida et al., 2018), histatins and derivatives (Mai et al., 2017), nisin (Su et al., 2018), LL-37 (da Silva et al., 2012), L-K6 (Sztukowska et al., 2019), and Bac8c (Ding et al., 2014), have been proven to show anti-caries effects without cytotoxicity. In our previous studies, the cationic AMP GH12 was proven to be effective as an anti-caries agent, which killed both planktonic and biofilms of cariogenic bacteria quickly and efficiently (Tu et al., 2016; Wang Y. et al., 2017; Jiang et al., 2018), suppressed the virulence factors of *S. mutans* (Wang et al., 2018a), and inhibited caries in a rat caries model (Wang et al., 2018b). GH12 could be a promising antimicrobial agent in oral application, but whether oral streptococci would be resistant to GH12 has not been evaluated.

The aim of this study was to explore the potential resistance of caries-related streptococci against *de novo* AMP GH12 through susceptibility assays to evaluate the intrinsic resistance of oral streptococci to GH12 and resistance assays with continuous GH12 selective pressure to explore the acquired resistance. As far as we know, although studies on anti-caries AMP are increasing, there have been few investigations into resistance to AMP until now. The present study, for the first time, evaluated the potential resistance of caries-related streptococci to GH12 before its clinical use in advance to prevent acquired GH12 resistance from emerging and transferring, which helps prolong the useful time of GH12 and provides adequate directions for its further clinical use.

## Materials and methods

### Peptides, chemicals, and assay kits

The peptide GH12 (Gly-Leu-Leu-Trp-His-Leu-Leu-His-His-Leu-Leu-His-NH_2_) was synthesized, identified, and purified to 98% by GL Biochem (Shanghai, China) as described previously (Wang Y. et al., 2017). The peptide was dissolved in sterile deionized water (DDW) and stored at −20 °C. Unless otherwise stated, the chemicals and assay kits were purchased from Sigma−Aldrich (St. Louis, MO, US).

### Bacterial strains and growth conditions

Caries-related streptococci (**Supplementary Table 1**) which included *S. mutans*, *S. gordonii* and *S. sanguinis*, were obtained from American Type Culture Collection (ATCC, Manassas, VA), Guangdong Culture Collection Center (GS, Guangzhou, China), and Japan Collection of Microorganisms (JCM, Tokyo, Japan). In addition, clinical strains of *S. mutans* (**Supplementary Table 1**) were included in the assay and were collected, isolated and identified according to a previous study from West China Hospital of Stomatology, Chengdu, China (Lu et al., 2015). All the strains were grown in brain-heart infusion broth (BHI; Oxoid, Basingstoke, Hampshire, UK) anaerobically (85% N_2_, 10% H_2_ and 5% CO_2_) at 37°C.

### Bacterial susceptibility assay

To evaluate the intrinsic resistance of different caries-related streptococci to antimicrobial agents, the minimal inhibitory concentration (MIC) was determined using a modified broth microdilution method (Wang Y. et al., 2017). BHI broth (80 µL), twofold serial dilutions of tested agents (20 μL), and bacteria suspension (100 µL) were placed in 96-well plates with a final concentration of bacteria of 1.0 × 10^8^ CFU/mL. The final concentrations of GH12 ranged from 4.0 to 128.0 mg/L. CHX and DAP (Marklin Biochemical Co., Ltd., Shanghai, China) served as controls. Negative and blank controls were incubated with DDW or fresh BHI broth, respectively. Incubation steps were based on the CLSI guidelines (CLSI, 2018). Absorbance at 600 nm (A_600_) was recorded using a microplate spectrophotometer (Multiskan GO; Thermo Scientific, USA). MIC was defined as the lowest concentration where no visible growth was observed (A_600(test)_/A_600(control)_ < 0.1). The tests were conducted and repeated for ten times on different days.

### Bacterial drug resistance assay

To further investigate whether caries-related streptococci could develop drug resistance to the tested antimicrobial agents, MIC measurements were performed following repeated serial passages, according to a previous method (Ikai et al., 2013). Aliquots (100 μL) of the bacterial suspensions in the sub-MIC well were taken and inoculated into 10 mL of fresh culture medium broth at 37°C anaerobically. The overnight bacterial suspensions were diluted to a concentration of approximately 1.0 × 10^8^ CFU/mL for the next passage MIC test. All MIC tests were repeatedly performed for 10 passages. After repeated exposures to one antimicrobial agent, an increase in the MIC value compared to the initial MIC indicated the acquisition of drug resistance by the bacterial cells. The tests were conducted and repeated for three times on different days.

### Assay of the stability of resistance

To evaluate the stability of acquired resistance, the assay was performed as previously described (Hong et al., 2016). Aliquots of resistant strain suspensions (100 µL) were taken and incubated in fresh 10 mL of BHI broth without antimicrobial agent under anaerobic conditions overnight, which was repeated for 10 continuous passages. And the bacterial susceptibility assay was performed at each passage to determine the MIC value. The tests were conducted and repeated for three times on different days.

### Cross-resistance assay

The susceptibility of the resistant strain to other antimicrobial agents was determined. The GH12-resistant *S. mutans* COCC33-14 isolate was selected and confirmed through the aforementioned assays. The resistant strain and parent strain were incubated anaerobically as mentioned before. Twofold serial dilutions of DAP and CHX were prepared to final concentrations ranging from 4.0 mg/L to 128.0 mg/L and 0.5 mg/L to 16.0 mg/L, respectively. The overnight suspension was adjusted to 2.0×10^8^ CFU/mL. Then, 100 μL of suspension, 80 μL of BHI broth, and 20 μL of prepared antimicrobial dilutions were mixed in each well of 96-well U-bottom microtiter plates. Negative and blank controls were incubated with DDW or fresh BHI broth, respectively. Incubation and determination are according to MIC measurement mentioned above in *Bacterial susceptibility assay*. The tests were conducted and repeated for three times on different days.

### Measurement of bacterial growth curves

Bacterial growth curves were generated following modified previous methods (Castillo et al., 2006) to evaluate the growth and metabolism changes of acquired resistant bacteria after being challenged with antibacterial agents. The initial concentrations of the bacteria (*S. mutans* UA159, *S. mutans* COCC33-14 parental strain, and GH12-resistant *S. mutans* COCC33-14 strain) were adjusted to 1 × 10^6^ CFU/mL. The inoculums were incubated at 37 °C anaerobically. A_600_ was detected over 24 h, and the growth inhibition kinetics were drawn based on the correspondence between A_600_ and incubation time. The tests were conducted and repeated for three times on different days.

### Whole genome sequencing and bioinformatics analysis

Genomic DNA was sequenced using a combination of Illumina NovaSeq6000 and Nanopore PromethION sequencing platforms. All of the analyses were performed using the online platform of Majorbio Cloud Platform (http://cloud.majorbio.com).

Glimmer Version 3.02 (Delcher et al., 2007) and GeneMarks (Besemer and Borodovsky, 2005) was used for codon sequence (CDS) prediction of chromosome and plasmid respectively. tRNA-scan-SE v2.0 (Chan and Lowe, 2019) was used for tRNA prediction, and Barrnap v0.9 was used for rRNA prediction. The predicted CDSs were annotated from NCBI’s nonredundant (NR), Swiss-Prot, Pfam, Gene Ontology (GO), Clusters of Orthologous Groups of proteins (COG), Kyoto Encyclopedia of Genes and Genomes (KEGG), Resfinder and Comprehensive Antibiotic Resistance Database (CARD). The detailed methods were presented in Supplementary S1.

### Statistical analysis

GraphPad Prism 8.0 (GraphPad, USA) was used to evaluate quantitative data statistically using one-way ANOVA and Fisher’s LSD tests, and differences were considered significant when P < 0.05.

## Results

### The resistance of caries-related streptococci and the stability of acquired resistance

To evaluate the spectrum of intrinsic resistance of caries-related streptococci, the total MIC values are shown in **Table 1**. The MICs of GH12 against *S. gordonii* and *S. sanguinis* were significantly higher than those against *S. mutans* (P < 0.05), indicating that *S. gordonii* and *S. sanguinis* possessed intrinsic resistance to GH12. Similarly, the MICs of DAP against *S. mutans* and *S. gordonii* were higher than those against *S. sanguinis* (P < 0.05), which indicated that DAP was more effective against *S. sanguinis*. However, the intrinsic resistance to CHX did not vary totally depending on species, and the MICs of two strains of *S. gordonii* (ATCC35105, ATCC33399) and *S. sanguinis* (JCM5708, SK36) against CHX were higher than those of *S. mutans* and *S. sanguinis* ATCC29667 (P < 0.05).

**Table 1:** MIC values of different species of streptococci against antimicrobial agents.

Strain	MIC (mg/L)*		
	GH12	CHX	DAP
*S. gordonii* ATCC35105	37.3 ± 13.1 a^*^	4.0 ± 0.0 b	42.7 ± 16.5 a
*S. gordonii* ATCC33399	37.3 ± 13.1 a	5.3 ± 2.1 a	42.7 ± 16.5 a
*S. gordonii* ATCC10558	32.0 ± 0.0 ab	3.7 ± 0.8 bc	42.7 ± 16.5 a
*S. sanguinis* SK36	26.7 ± 8.3 b	4.7 ± 1.6 ab	10.7 ± 4.1 bc
*S. sanguinis* ATCC29667	21.3 ± 8.3 bc	2.7 ± 1.0 c	10.7 ± 4.1 bc
*S. sanguinis* JCM5708	16.0 ± 0.0 c	4.7 ± 1.6 ab	5.3 ± 2.1 c
*S. mutans* ATCC25175	10.7 ± 4.1 cd	2.3 ± 0.8 c	18.7 ± 6.5 b
*S. mutans* GS-5	9.3 ± 3.3 d	2.3 ± 0.8 c	18.7 ± 6.5 b
*S. mutans* UA159	8.0 ± 0.0 d	2.0 ± 0.0 c	16.0 ± 0.0 b

* Data are presented as means ± standard deviations (n=10). Different letters from a to d indicate significant differences (P < 0.05).

**Figure 1:**
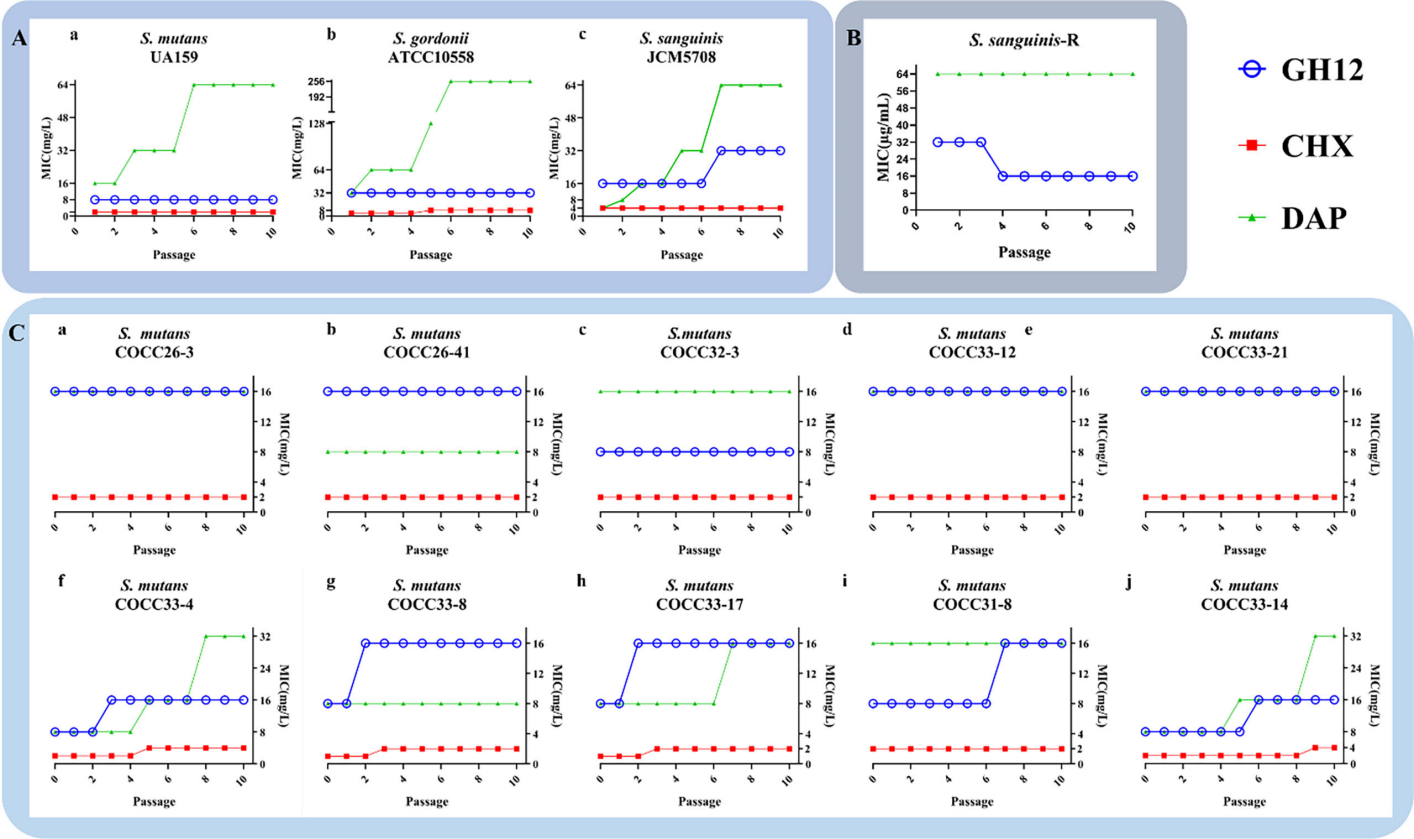
**(A)** MICs of GH12, CHX and DAP against *S. mutans* UA159 **(a)**, *S. gordonii* ATCC10558 **(b)** and *S. sanguinis* JCM5708 **(c)** from passages 1 to 10. **(B)** Evaluation of the stability of acquired resistance of *S. sanguinis*-R strain. *S. sanguinis*-R: resistant *S. sanguinis* strain toward GH12 and DAP. **(C)** MICs of GH12, CHX and DAP against *S. mutans* clinical strains (passage 0) and MICs after each passage under exposure to sub-MIC concentration (passage 1 to 10). The data are represented as mean ± standard deviation (n=3).

To evaluate the spectrum of GH12 acquired resistance in three different species of streptococci, the serial MICs of *S. mutans* UA159, *S. gordonii* ATCC10558 and *S. sanguinis* JCM5708 are presented in **Figure 1A**. MIC values of GH12 did not increase except *S. sanguinis*, from 16.0 mg/L to 32.0 mg/L at passage 7. In addition, only *S. gordonii* ATCC10558 developed resistance to CHX through continuous passages, and the MIC value increased from 4.0 mg/L to 8.0 mg/L at passage 5. Unexpectedly, the MIC of DAP against all of the tested strains increased and even to high-level drug resistance for *S. gordonii* (from 32.0 mg/L to 128.0 mg/L) and *S. sanguinis* (from 4.0 mg/L to 64.0 mg/L).

Nevertheless, the MIC of GH12-resistant *S. sanguinis* declined back to the initial MIC value of the parental strain after continuous passages in fresh BHI broth (**Figure 1B**). In contrast, the DAP-resistant *S. sanguinis* strain maintained an increased MIC value (**Figure 1B**). These results indicated that *S. sanguinis* developed stable resistance to DAP but transient resistance to GH12.

### The resistance of *S. mutans* clinical strains and the stability of acquired resistance

The total MIC values of ten *S. mutans* clinical strains of are shown in **Figure 1C** (passage 0). The different clinical isolates of *S. mutans* showed varied susceptibilities, which demonstrated different potential drug resistances. To further evaluate the spectrum of GH12 acquired resistance in different isolates of *S. mutans*, MICs against ten *S. mutans* clinically isolated strains from passages 1 to 10 were monitored. As shown in **Figure 1C** (passage 1-10), among all ten tested strains, five clinically isolated strains (*S. mutans* COCC26-3, COCC26-41, COCC32-3, COCC33-12, COCC33-21) maintained the same MIC values under the continuous selective burden of all antimicrobial agents. For the other clinical isolates, a tendency of acquired resistance was observed through the phenomenon of increased MIC of GH12 along the repeated passages. The MIC values of *S. mutans* COCC33-8, COCC33-4, and COCC33-17 increased twofold at passage 2 or 3, and COCC31-8 and COCC33-14 increased at passage 6 or 7. On the other hand, the MICs of CHX increased twofold against four isolates of *S. mutans* (COCC33-8, COCC33-4, COCC33-17 and COCC33-14). In addition, the MICs of DAP against three isolates (COCC33-4, COCC33-17 and COCC33-14) increased by 2-4 times.

**Figure 2:**
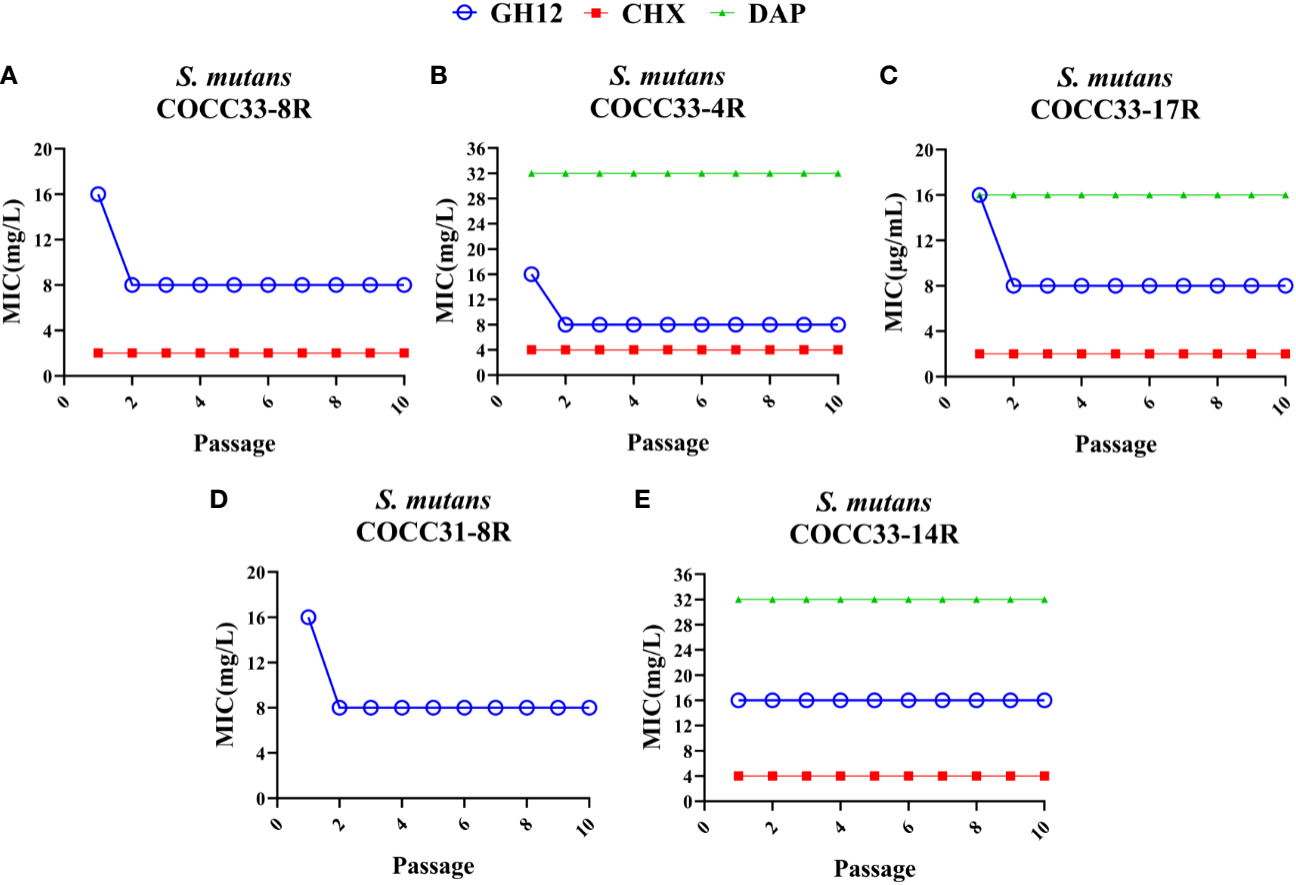
Evaluation of the stability of acquired resistance of *S. mutans* clinical strains. MIC values of GH12, CHX and DAP to *S. mutans* COCC33-8R **(A)**, COCC33-4R **(B)**, COCC33-17R **(C)**, COCC31-8R **(D)** and COCC33-14R **(E)** after each passage regrown in the lack of antimicrobial agent, respectively (mean ± SD; n = 3). R, resistant strain.

To test the stability of acquired resistance, resistant strains were regrown without antimicrobial agents for 10 passages, and the MIC values were determined. As shown in **Figure 2**, although the MICs of the five isolates decreased, only GH12-resistant *S. mutans* COCC33-14 maintained an increased MIC value. In contrast, all of the CHX- or DAP-resistant strains retained the increased MIC values, which were 2-4 times higher than those of the parental strains. The unchanged MICs demonstrated that these isolates developed stable resistance to the corresponding antimicrobial agent.

### The cross-resistance and kinetic growth curves of the GH12-resistant strain

The MIC values of other antimicrobial agents against GH12-resistant *S. mutans* COCC33-14 strain (COCC33-14R) are shown in **Table 2**. For CHX, the MIC value was 2.0 mg/L and equal to the parental strain, which indicated that COCC33-14R was susceptible to CHX. However, the MIC of DAP against COCC33-14R was four times higher than the that of parental strain. The results demonstrated that COCC33-14R showed cross-resistance to DAP but no cross-resistance to CHX.

**Table 2:** Cross-resistance of GH12-resistant *S. mutans* COCC33-14 to CHX and DAP.

Bacteria strains	MIC (mg/L)^a^		
	GH12	CHX	DAP
COCC33-14	8.0 ± 0.0	2.0 ± 0.0	8.0 ± 0.0
COCC33-14R^b^	16.0 ± 0.0	2.0 ± 0.0	32.0 ± 0.0

a: Data are represented as mean ± standard deviation (n=3).

b: GH12-resistant S. mutans COCC33-14.

**Figure 3:**
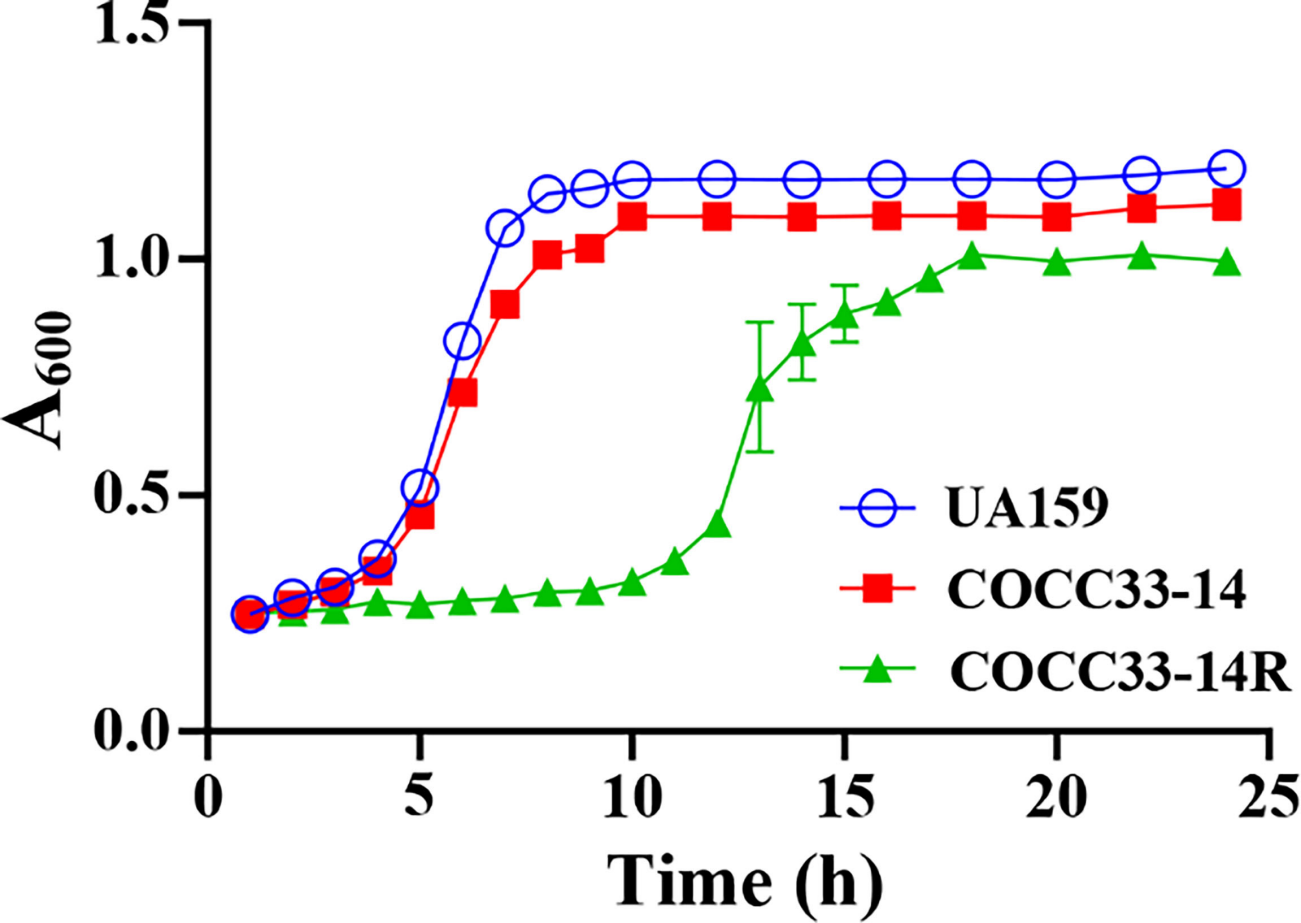
Kinetic growth curves (mean ± SD; n = 3). UA159: *S. mutans* UA159; COCC33-14: *S. mutans* COCC33-14 parental strain; COCC33-14R: GH12-resistant *S. mutans* COCC33-14 strain.

To compare the growth differences between UA159, COCC33-14, and COCC33-14R, 24-h representative growth curves are shown in **Figure 3**. UA159 and COCC33-14 showed similar lag phases and log phases, of which lag phase ended at the fourth hour, and the log phase continued from the fifth to eighth hour. Otherwise, COCC33-14R had a much longer lag phase, which sustained almost 11 h. Additionally, although with no statistical significance, the stationary phase of COCC33-14R was slightly lower than that of the other two strains. The results indicated that the growth metabolism of COCC33-14R was delayed compared to that of the parental strain.

### Whole genome sequencing analysis

**Figure 4:**
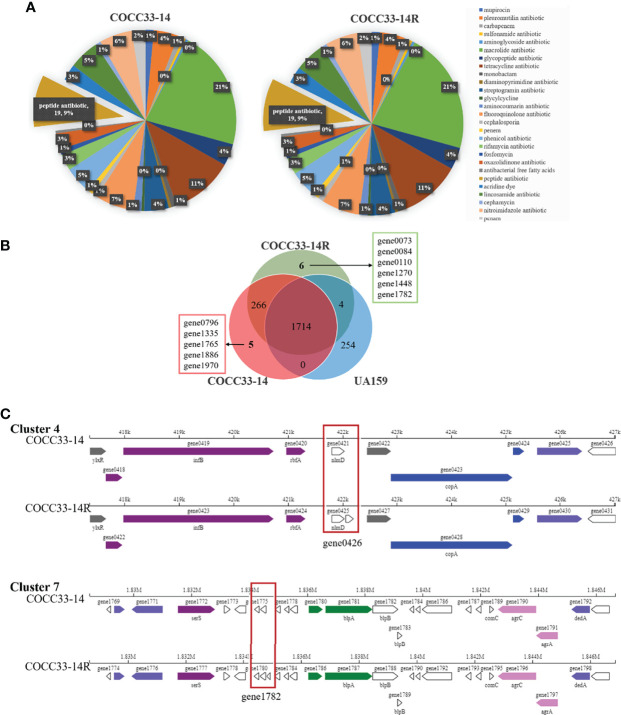
Analysis of genome of COCC33-14R. **(A)** CARD annotations and prediction of probable resistant genes. **(B)** Differential genes between COCC33-14R and COCC33-14. **(C)** Comparison of linear plot of secondary metabolite gene Cluster 4 and Cluster 7.

The whole genomes of COCC33-14R, COCC33-14 and UA159 were sequenced, assembled, predicted and annotated, followed by comparison and analysis. All three strains have only one chromosomal DNA, and no plasmid, of which COCC33-14R possesses the longest sequences and most CDSs (**Supplementary Table 2**). Resfinder database analysis showed that no resistance genes were found in any strain. CARD annotations and predictions of probable resistance genes of COCC33-14R and COCC33-14 are shown in **Figure 4A**, and no differences were found between them.

The differential genes between COCC33-14R and COCC33-14 are shown in a Venn diagram (**Figure 4B**). COCC33-14R acquires 6 new genes and misses 5 genes compared with COCC33-14. According to the annotations of the 11 differential genes (**Supplementary Table 3**), most of them encode hypothetical proteins. Only GO annotation of one new gene (gene1782|COCC33-14R) shows that it’s related to integral component of membrane. One missing gene of COCC33-14R (gene1886|COCC33-14) is related to the biogenesis and function of ribosomal protein S14.

The CDSs of carbohydrate-active enzymes (CAZymes) and secondary metabolites were analyzed. No difference in CAZymes was found between COCC33-14R and COCC33-14. However, there was one more gene in secondary metabolite gene Cluster 4 and Cluster 7 of COCC33-14R than COCC33-14, which were gene0426 and gene1782, respectively (**Figure 4C**). The annotation of gene1782 is as mentioned above, and gene0426 has no annotation.

## Discussion

AMP has been used widely in the clinic, and systemic administration of AMP causes resistance in oral streptococci (Fernandez and Hancock, 2012; Moffatt et al., 2019). According to the Center for Disease Control of the USA, novel antibiotics are far from sufficient, and appropriate use of antibiotics could reduce the emergence of resistance (CDC, 2019). As a result, it’s necessary to study the potential resistance of oral bacteria to GH12 in advance to improve its clinical use. The present study explored the intrinsic and acquired drug resistance of caries-related streptococci toward the *de novo* AMP GH12 for the first time, and further investigated the probable mechanism of acquired resistance to GH12. The results of the current study demonstrated that different species of oral streptococci showed different intrinsic and acquired resistance to GH12. More health-associated strains, *S. gordonii* and *S. sanguinis* were intrinsically resistant to GH12, while more cariogenic strain, *S. mutans* showed different susceptibilities to GH12. In addition, the frequency of GH12 resistance in these oral streptococci was low. Among all thirteen tested caries-related streptococci in this study, only one *S. mutans* clinical strain (COCC33-14) developed stable resistance to GH12. GH12 induced transient resistance in *S. sanguinis* JCM5708 and four isolates of *S. mutans* clinical strains and no resistance in the other seven streptococci. However, DAP and CHX always selected stable resistant isolates. Furthermore, the stability of acquired resistance was tested after resistance assays. In addition, the new gene in GH12-resistant strain COCC33-14R might be related to integral component of membrane.

Streptococci are one of the most predominant bacteria in oral biofilms, and are associated with biofilm colonization and plaque formation (Lee and Lee, 2019). *S. mutans* is considered a representative cariogenic bacterium, with the virulence factors of acidurance, acidogenicity, and the ability to produce glucans to adhere to tooth surface (Suppiger et al., 2020). On the other hand, *S. sanguinis* and *S. gordonii* have the ability not only to produce acid but also to produce alkali (Huang et al., 2012) and H_2_O_2_ (Cheng et al., 2020), which are antagonistic with *S. mutans* (Huang et al., 2018) and partly linked to protection against dental caries. The results in the present study demonstrate that these bacteria are intrinsically resistant to GH12 (**Table 1**), so GH12 could have the advantage of being able to suppress *S. mutans* more selectively *in vivo*. This is consistent with our previous study, where GH12 could selectively inhibit *S. mutans* in a three-species biofilm (Jiang et al., 2018). However, the previous study neither looked into the aspect of intrinsic resistance nor further studied the acquired resistance. Remarkably, one *S. sanguinis* strain JCM5708 and four *S. mutans* clinical strains (COCC33-4, COCC33-8, COCC33-17, COCC31-8) showed transient resistance to GH12, which was reversed after several passages without GH12 (**Figures 1B, C**, **2A–D**). A similar phenomenon was also observed in nisin-resistant *Listeria monocytogenes*, and its meaning was unclear (Gravesen et al., 2002). Our hypothesis was that the phenomenon of transient resistance might indicate temporary phenotypic changes rather than genetic mutation, which help bacteria resist the antibiotic effect for a short time (Andersson and Hughes, 2010). However, further studies are needed.

Since *S. mutans* is associated with increased caries risk and poor caries outcomes (Lima et al., 2020), and the emergence of *S. mutans* resistance is undesirable for caries treatment and prevention, further studies on different *S. mutans* strains were conducted. Different strains of the same bacterium have shown a great degree of heterogeneity. It has been reported that different clinical isolates have diverse susceptibilities to the same antimicrobial agent, such as silver nanoparticles (Espinosa-Cristóbal et al., 2012) and chitosan (Pasquantonio et al., 2008). In the present study, *S. mutans* clinical isolates were collected and identified by morphological identification, VITEK automatic microorganism identification and 16S rRNA gene sequencing and were named according to human oral microbiome database and EzTaxon server 2.1 (Lu et al., 2015). The clinical strains of *S. mutans* showed various intrinsic and acquired resistance to GH12 (**Figure 1C**), consistent with the results of other studies (Espinosa-Cristóbal et al., 2012). The mechanism may be not only species-dependent but also serotype-dependent, but further studies are needed.

CHX resistance in streptococci was found early in the 1970s (Schiøtt and Löe, 1972), but it has not raised much attention. Afterwards, there were several reports about CHX resistance in oral streptococci, such as *S. sanguinis* (Westergren and Emilson, 1980), *S. gordonii* (Wang S. et al., 2017) and *S. mutans* ATCC20523 (Verspecht et al., 2019). The present study also showed increased MIC values in *S. gordonii* ATCC35105 and four clinical isolates of *S. mutans* to CHX (**Figure 1A, b**, **Figure 1C, f-h, j**). The CHX resistance would be detrimental to infectious control and a unique clone of community-acquired MRSA was found to expand according to the acquisition of a plasmid consisting of genes (*qacA/B*) conferring resistance to CHX (Copin et al., 2019). CHX resistance could induce resistance in clinical *Klebsiella pneumoniae* isolates and cross-resistance to colistin (Wand et al., 2017). The cross resistance of CHX-resistant strains has also been well documented (Kampf, 2018a; Kampf, 2019). The present study could provide more evidence for acquired CHX resistance in *S. mutans* clinical strains *in vitro*. DAP is a cyclic lipopeptide antibiotic that has bactericidal activity against staphylococci and streptococci and is less renal toxicity than vancomycin (Larioza et al., 2011). Thus, DAP has been a best alternative for the therapy of multidrug-resistant streptococci infectious diseases for nearly 20 years. In our study, DAP showed the ability to select for resistant strains in three tested species of caries-related streptococci (**Figure 1A**), and three of clinical isolates of *S. mutans* (**Figure 1C, f, h, j**). In addition, *S. gordonii* and *S. sanguinis* rapidly developed high-level resistance to DAP. This was the same situation as other studies, in which streptococci acquired DAP resistance *in vitro* and *in vivo* after exposure to DAP (Garcia-de-la-Maria et al., 2013; Parrett et al., 2020). Another study also observed that stable and high-level DAP resistant mutants in *S. mitis-oralis* were produced by serial passages in sublethal DAP *in vitro* (Mishra et al., 2020). A variety of bacteria colonize the oral cavity and infect not only oral health but also general health. Oral bacteria can enter the bloodstream through ulcerated inflamed crevices and pocket epithelium and adjacent gingival microcirculation during surgery or during routine brushing and chewing, leading to systemic disease (Dhotre et al., 2016). Both previous studies and our results indicated that GH12 is advantageous over the controls because of its lower frequency of stable resistance.

The mechanism of CHX resistance is not clear, but it is widely believed to be related to changes in efflux pumps and cell membranes (Kampf, 2018b). As an AMP, the mechanism of DAP resistance in Gram-positive bacteria is generally considered to be associated with important adaptive changes in the cell wall and cell membrane homeostasis (Tran et al., 2015). However, based on CARD annotations and prediction of probable resistance genes (**Figure 4A**), the GH12-resistant strain did not possess any known resistance genes, and Resfinder database analysis did not find any resistance gene in any strain, suggesting that there is no similarity between the GH12-resistant and other known resistance genomes. As a result, the mechanism of GH12 resistance has to be discussed in the other aspects as follows.

The phenotypic consequences of GH12 resistance were evaluated with particular reference to antibiotic growth curves and cross-resistance, which, together with the results of gene sequencing and analysis, provide clues to elucidate the mechanism of GH12 resistance. First, GH12-resistant *S. mutans* COCC33-14 showed cross-resistance to DAP but was still susceptible to CHX (**Table 2**), suggesting that the mechanism of GH12 resistance is related to similarities and differences in the antimicrobial mechanisms of GH12, DAP and CHX. The first step of the antimicrobial mechanisms of GH12, DAP and CHX is thought to be binding to bacterial surface by the action of electric charge in all cases. However, the next interaction of disruption of bacterial cells followed by binding is different, which may determine the potential for resistance development (Gilbert and Moore, 2005). CHX consists of two cationic groups and one hydrophobic bridging structure, and the hydrophobic region is 6 carbons long and inflexible and incapable of folding sufficiently to interdigitate into the bilayer (Gilbert and Moore, 2005). In contrast, GH12 and DAP are both AMPs and have similar antimicrobial targets, phospholipid bilayers, and their second step of bactericidal action is believed to form pores on the cell membrane (Wang Y. et al., 2017; Heidary et al., 2018). On the other hand, WGS analysis also demonstrated that the new gene of COCC33-14R (gene1782) is related to integral components of the membrane. According to the two pieces of evidence above, the resistance of COCC33-14R was probably associated with changes in the cell envelope, and major differences in antimicrobial mechanisms between AMPs and CHX. Second, the metabolism of the GH12-resistant strain was reduced after continuous exposure to GH12. Antibiotic resistance is always associated with fitness cost, which is typically observed as a reduced bacterial growth rate (Andersson and Hughes, 2010), which is consistent with the present study. In a recent study, DAP-resistant *S. mitis* also possessed a decreased growth rate and a decreased growth biomass compared to DAP-susceptible *S. mitis* (Parrett et al., 2020). WGS demonstrated that the missing gene in COCC33-14R (*rpsN*) was related to bacterial metabolism and growth and commonly existed in many bacteria, including streptococci (Makthal et al., 2020), and the absence of *rpsN* could result in a defective 30S subunit of the ribosome and then affect translation (Nanamiya and Kawamura, 2010). Although the CDS, which encodes a hypothetical protein, needs further evaluation, based on this explicable evidence, we deduced that the consecutive sublethal GH12 pressure selected for the gene1782 mutation, to change the cell envelope of COCC33-14R to induce its resistance, and in the meantime, the *rpsN* was missing in COCC33-14R’s genome to increase its fitness cost. However, further investigations are needed, such as thickness, permeability and specific cell membrane components of COCC33-14R. Additionally, further studies on metabolomics, transcriptomics, and genomics are necessary to understand the connection between this type of resistance and metabolic changes.

In conclusion, stable resistant mutants of caries-related streptococci could hardly be selected by exposure to consecutive sublethal GH12, but the risk still exists. The resistance mechanism of COCC33-14R is probably related to changes in the cell envelope. To avoid the emergence of GH12 resistance, more studies with different variables, such as selection methods, concentrations and drug treatment times are needed.

## Data availability statement

The data presented in the study are deposited in the Genome Database of NCBI (https://www.ncbi.nlm.nih.gov/genome/) repository, and are under BioProject ID PRJNA862258 with accession numbers: CP101984 (Streptococcus mutans UA159), CP101985 (Streptococcus mutans COCC33-14R) and CP101986 (Streptococcus mutans COCC33-14).

## Author contributions

XL: The conception and design of the study, acquisition of data, analysis and interpretation of data, drafting the article. YW: The conception and design of the study, acquisition of data, analysis and interpretation of data. XJ: Acquisition of data. YZ: Acquisition of data. XZ: Analysis and interpretation of data. JW: Analysis and interpretation of data. NT: Analysis and interpretation of data. LZ: The conception and design of the study, analysis and interpretation of data. All authors contributed to revise it critically for important intellectual content and approve the final version of the manuscript. All authors contributed to the article and approved the submitted version.

## Funding

This work was supported by the National Natural Science Foundation of China (grant number 81970931).

## Conflict of interest

The authors declare that the research was conducted in the absence of any commercial or financial relationships that could be construed as a potential conflict of interest.

## Publisher’s note

All claims expressed in this article are solely those of the authors and do not necessarily represent those of their affiliated organizations, or those of the publisher, the editors and the reviewers. Any product that may be evaluated in this article, or claim that may be made by its manufacturer, is not guaranteed or endorsed by the publisher.
