# Supplementary material for: Investigation of drug resistance of caries-related streptococci to antimicrobial peptide GH12
Source: Front Cell Infect Microbiol. 2022 Sep 8;12:991938. doi: 10.3389/fcimb.2022.991938 (PMC9492880; doi:10.3389/fcimb.2022.991938)
Supplement: Supplementary file 1 [file Table_1.docx]

Supplement Supplementary Material

**Supplementary Table 1 Bacteria tested and their sources**

| **Strain** | **Source and/or reference** | **Clinical isolated strains** | **Source and/or reference** | |
| --- | --- | --- | --- | --- |
| *S. mutans* UA159 | American Type Culture Collection | *S. mutans* COCC32-3 | West China Hospital of Stomatology(Lu W et al., 2015) | |
| *S. mutans* GS-5 | Guangdong Culture Collection Center | *S. mutans* COCC33-17 | West China Hospital of Stomatology | |
| *S. mutans* ATCC25175 | American Type Culture Collection | *S. mutans* COCC31-8 | West China Hospital of Stomatology | |
| *S. gordonii* ATCC35105 | American Type Culture Collection | *S. mutans* COCC33-8 | West China Hospital of Stomatology | |
| *S. gordonii* ATCC10558 | American Type Culture Collection | *S. mutans* COCC26-3 | West China Hospital of Stomatology | |
| *S. gordonii* ATCC33399 | American Type Culture Collection | *S. mutans* COCC33-12 | West China Hospital of Stomatology | |
| *S. sanguinis* JCM5708 | Japan Collection of Microorganisms | *S. mutans* COCC33-4 | West China Hospital of Stomatology | |
| *S. sanguinis* SK36 | American Type Culture Collection | *S. mutans* COCC26-41 | West China Hospital of Stomatology | |
| *S. sanguinis* ATCC29667 | American Type Culture Collection | *S. mutans* COCC33-14 | West China Hospital of Stomatology | |
|  |  | *S. mutans* COCC33-21 | West China Hospital of Stomatology |  |

# S1 *Whole genome sequencing and bioinformatics analysis*

1. ***Library construction and genome sequencing***

Genomic DNA was sequenced using a combination of Illumina NovaSeq6000 and Nanopore PromethION sequencing platforms. Illumina data were used to evaluate genome complexity and accuracy. For Illumina sequencing, at least 1 μg of genomic DNA was used for each strain in sequencing library construction. DNA samples were sheared into 400~500-bp fragments using an M220 Focused Acoustic Shearer (Covaris, MA, USA) following the manufacturer’s protocol. Illumina sequencing libraries were prepared from the sheared fragments using the NEXTflex™ Rapid DNA-Seq Kit. Briefly, 5’ ends were first end-repaired and phosphorylated. Next, the 3’ ends were A-tailed and ligated to sequencing adapters. The third step is to enrich the adapters-ligated products using PCR. The prepared libraries then were used for paired-end Illumina sequencing (2 × 150 bp) on an Illumina NovaSeq6000 platform (Illumina, San Diego, CA, USA). For Nanopore sequencing, approximately 10 μg of genomic DNA was isolated and size-select using BluePippin (Sage Science, Beverly, MA, USA) and processed according to the (1D) Ligation Sequencing Kit (SQK-LSK109) protocol. Briefly, DNA fragments were repaired using NEBNext FFPE Repair Mix (New England Biolabs). After end-repair and 3’-adenylation with the NEBNext End repair/dA-tailing Module reagents (New England Biolabs), then attach sequencing adapters supplied in the SQK-LSK109 kit to the DNA ends. The final library was loaded onto R9.4 flow cell using the PromethION DNA sequencer (Oxford Nanopore, Oxford, UK) for 48 hours.

1. ***Gene assembly and annotation***

The data generated from Nanopore and Illumina platform were used for bioinformatics analysis. All of the analyses were performed using the free online platform of Majorbio Cloud Platform (http://cloud.majorbio.com) from Shanghai Majorbio Bio-pharm Technology Co.,Ltd. The detailed procedures are as follows. The raw Illumina sequencing reads generated from the paired-end library were subjected to quality-filtered using fastp v0.23.0. Nanopore reads were extracted, basecalled and demultiplexed, and trimmed using ONT Guppy with the minimum Q score cutoff of 7. Then the clean short and long reads were co-assembled to construct complete genomes using Unicycler -0.4.7(Wick et al., 2017). As a final step, Unicycler uses Pilon v1.22 to polish the assembly using short-read alignments, reducing the rate of small errors.

Glimmer Version 3.02(Delcher et al., 2007) and GeneMarks(Besemer & Borodovsky, 2005) was used for codon sequence (CDS) prediction of chromosome and plasmid respectively. tRNA-scan-SE v2.0(Chan & Lowe, 2019) was used for tRNA prediction, and Barrnap v0.9 was used for rRNA prediction. The predicted CDSs were annotated from NCBI's nonredundant (NR), Swiss-Prot, Pfam, Gene Ontology (GO), Clusters of Orthologous Groups of proteins (COG), Kyoto Encyclopedia of Genes and Genomes (KEGG), Resfinder and Comprehensive Antibiotic Resistance Database (CARD).

**Supplementary Table 2 Statistics of genomic analysis**

| **Bacteria Strains** | **Genome Size (bp)** | **Chrom No**. | **Plas No.** | **GC Content(%)** | **Gene No.** |
| --- | --- | --- | --- | --- | --- |
| COCC33-14R | 2115009 | 1 | 0 | 36.83 | 1990 |
| UA159 | 2033272 | 1 | 0 | 36.83 | 1972 |
| COCC33-14 | 2114814 | 1 | 0 | 36.83 | 1985 |

**Supplementary Table 3 Annotations of differential genes**

| BacteriaStrain | Gene ID | Gene Name | Gene Description | | | | | |
| --- | --- | --- | --- | --- | --- | --- | --- | --- |
|  |  |  | NR | Swiss-Prot | Domain | COG | GO | KO |
| COCC  33-14R | gene0073 | - | hypothetical protein | - | - | - | - | - |
|  | gene0084 | - | hypothetical protein | - | - | - | - | - |
|  | gene0110 | - | hypothetical protein | - | - | - | - | - |
|  | gene1270 | - | hypothetical protein | - | - | - | - | - |
|  | gene1448 | - | hypothetical protein | - | - | - | - | - |
|  | gene1782 | - | hypothetical protein | - | - | - | integral component of membrane | - |
| COCC  33-14 | gene0796 | - | hypothetical protein | - | - | - | - | - |
|  | gene1335 | - | hypothetical protein | - | - | - | - | - |
|  | gene1765 | - | hypothetical protein | - | - | - | - | - |
|  | gene1886 | rpsN | MULTISPECIES: type Z 30S ribosomal protein S14 | 30S ribosomal protein S14 | Ribosomal protein S14p/S29e | Binds 16S rRNA, required for the assembly of 30S particles and may also be responsible for determining the conformation of the 16S rRNA at the A site (By similarity) | translation; ribosome; structural constituent of ribosome; rRNA binding; zinc ion binding | small subunit ribosomal protein S14 |
|  | gene1970 | - | hypothetical protein | - | - | - | - | - |

***Reference***

Besemer, J., and Borodovsky, M. (2005). GeneMark: web software for gene finding in prokaryotes, eukaryotes and viruses. *Nucleic Acids Res*, *33*(Web Server issue), W451-454. [doi:10.1093/nar/gki487](https://doi.org/10.1093/nar/gki487)

Chan, P. P., and Lowe, T. M. (2019). tRNAscan-SE: Searching for tRNA Genes in Genomic Sequences. *Methods Mol Biol*. 1962:1-14. [doi: 10.1007/978-1-4939-9173-0_1](https://doi.org/10.1007/978-1-4939-9173-0_1)

Delcher, A. L., Bratke, K. A., Powers, E. C., and Salzberg, S. L. (2007). Identifying bacterial genes and endosymbiont DNA with Glimmer. *Bioinformatics*, *23*(6), 673-679. [doi: 10.1093/bioinformatics/btm009](https://doi.org/10.1093/bioinformatics/btm009)

Lu W, Wu F, Zhou X, Wu L, Li M, Ren B, et al. (2015). Isolation and identification of aerobic and facultative anaerobic bacteria in the oral cavity. *Nan Fang Yi Ke Da Xue Xue Bao*, *35*, 1710–1714. [doi: 10.3969/j.issn.1673-4254.2015.12.09](https://doi.org/10.3969/j.issn.1673-4254.2015.12.09)

Wick, R. R., Judd, L. M., Gorrie, C. L., and Holt, K. E. (2017). Unicycler: Resolving bacterial genome assemblies from short and long sequencing reads. *PLoS Comput Biol*, *13*(6), e1005595. [doi: 10.1371/journal.pcbi.1005595](https://doi.org/10.1371/journal.pcbi.1005595)
